# A time-resolved immunoassay to measure serum antibodies to the rotavirus VP6 capsid protein

**DOI:** 10.1016/j.jviromet.2012.11.003

**Published:** 2013-04

**Authors:** Owen Kavanagh, Xi-Lei Zeng, Sasirekha Ramani, Indrani Mukhopadhya, Sue E. Crawford, Gagandeep Kang, Mary K. Estes

**Affiliations:** a Department of Molecular Virology and Microbiology, Baylor College of Medicine, Houston, TX 77030, USA; b Department of Gastrointestinal Sciences, Christian Medical College, Vellore, TN 632004, India

**Keywords:** Rotavirus, Recombinant VP6, DELFIA, ELISA

## Abstract

The rotavirus (RV) inner capsid protein VP6 is widely used to evaluate immune response during natural infection and in vaccine studies. Recombinant VP6 from the most prevalent circulating rotavirus strains in each subgroup (SG) identified in a birth cohort of children in southern India [SGII (G1P[8]) and SGI (G10P[11])] were produced. The purified proteins were used to measure VP6-specific antibodies in a Dissociation-Enhanced Lanthanide Fluorometric Immunoassay (DELFIA). The ability of the assay to detect a ≥2 fold rise in IgG level in a panel of serum samples from a longitudinal study was compared to a gold standard virus-capture ELISA. A strong association was observed between the assays (*p* < 0.001; chi-squared test) with assay performances remaining similar when the samples were subdivided as having a fold change increase in VP6 antibody levels (a) within 90 days of RV RNA detection in stool or (b) if no RV RNA was detected within that time period. This study demonstrates the suitability of using recombinant proteins to measure anti-RV immune responses and serves as a “proof of principle” to examine the antibody responses generated to other recombinant RV proteins and thereby possibly identify a correlate of protection.

## Highlights

► Two recombinant VP6 capsid proteins from an Indian birth cohort were expressed. ► The proteins were used to establish a time-resolved fluorescence DELFIA. ► DELFIA was compared to a gold standard ELISA using cohort serum samples. ► A significant association was observed between the two assays (*p* < 0.05). ► This demonstrates recombinant proteins can be used to study RV antibody responses.

Group A rotaviruses (RV) are the major cause of severe gastroenteritis in infants and young children worldwide resulting in an estimated 453,000 deaths per annum in 2008 before the introduction of the universal rotavirus vaccination programs (Tate et al., 2012). India alone accounted for 22% of worldwide deaths with an estimated 98,621 children dying from RV diarrhea in that year (Tate et al., 2009) and in a recent study of all causative agents, RV was identified as the most common cause of disease in children hospitalized with diarrhea in southern India (Ajjampur et al., 2008). The mechanisms and effectors of protection against RV infection are not completely understood. To date, efforts to elucidate any protective marker have focused mainly on studying the immune responses to the intact virus capsid. Attempts have been made to examine antibodies generated to the individual viral proteins but these studies have not used formats that can be employed in large scale studies (Ishida et al., 1996; Johansen et al., 1994; Richardson and Bishop, 1990; Svensson et al., 1987).

Classification of RV into groups and subgroups is based on the major capsid protein VP6 (Greenberg et al., 1983; Estes and Cohen, 1989). Group A RVs are characterized into 4 subgroups (SG) denoted SGI, II, I/II and non-I/II with the majority of mammalian strains classified as SGI or II. VP6 is an immundominant antigen with high sequence homology and common antigenic epitopes amongst group A RV (Estes and Cohen, 1989). This makes VP6 a widely used antigenic target in clinical and seroepidemiological studies and serum immunoglobulin responses against VP6 detected by ELISA is regarded as an indicator of RV immunity, including vaccine take (Ward et al., 1989).

The purpose of this study was to compare the efficiency of a recombinant VP6-based Dissociation-Enhanced Lanthanide Fluorometric Immunoassay (DELFIA) with a gold standard virus-capture ELISA to detect serum IgG to VP6. The production of SGI and SGII recombinant VP6 (rVP6) antigen from circulating RV strains identified in a birth cohort of children from an urban slum in southern India is described. A panel of serum samples from this longitudinal study was used to compare the recombinant antigen-based DELFIA to the virus-capture ELISA.

A birth cohort of 373 children from urban slums in Vellore, India, was followed-up as part of a study on natural course of rotavirus disease and infections. The methods for recruitment and follow-up of the cohort have been published previously (Gladstone et al., 2008, 2010, 2011). Stool samples collected every fortnight for surveillance and during all diarrheal episodes were screened for rotavirus using a commercial enzyme immunoassay (Rotavirus IDEIA, Dako). Serum samples were collected at least every six months and within 4 weeks of a rotavirus diarrhea whenever possible. However, for more sensitive detection of rotavirus in stool, every stool sample from a subset of 20 children nested within the cohort was tested by PCR. These 20 children experienced a total of 108 diarrheal episodes (median = 4.5 episodes) and 40 rotavirus infections (median = 2 rotavirus infections) including 29 symptomatic and 11 asymptomatic infections based on the definition of a sample considered positive for rotavirus either based on two ELISA tests or an RT-PCR assay as described previously (Gladstone et al., 2011). A total of 236 serum samples from these 20 children were tested in this study. Apart from 6 monthly samples, the median time of sample collection post rotavirus infection was 40 days (interquartile range 32–52 days).

Viral RNA extraction, cDNA synthesis and RV genotyping was carried out as described previously (Banerjee et al., 2006). The sub-grouping of the rotavirus using VP6 was performed for samples from both genotypes as described previously (Iturriza Gomara et al., 2002). Briefly, amplification of the gene encoding the VP6 protein was carried out using the oligonucleotide primers VP6-F (5′-GACGGVGCRACTACATGGT-3′; nt 747–766) and VP6-R (5′-GTCCAATTCATNCCGGTGG-3′; nt 1126–1106) which amplifies a 379 bp region encoding the SG epitope sequences and the trimerization region. This amplicon was sequenced using the same primers and then assigned to a SG based on phylogenetic analysis.

Overall, the most common genotype identified in this population was G1P[8] while G10P[11] was the most common cause of infections in the neonatal period (Gladstone et al., 2011; Banerjee et al., 2007). Nucleotide sequence analysis of the cloned gene 6 cDNA of these two strains revealed that the VP6 gene segments encoded a single open reading frame (ORF) that was 1191 bp long but only displayed 80% nt and 91% amino acid sequence homology (data not shown), between the VP6 of the G1P[8] strain belonging to SGII and that of G10P[11] to SGI. Both ORFs encoded predicted proteins consisting of 397 amino acid (AA) with a molecular weight of 44.9 kDa. The VP6 antigen derived from rotavirus strain WC3 used in the ELISA belongs to SGI (Ward et al., 1989) and shares a predicted AA homology of 98% and 91% for VP6 SGI and SGII, respectively.

Genes amplified from representative human G1P[8] strain (SGII) and a human-bovine reassortant G10P[11] (SGI) VP6 from the birth cohort were chosen to produce recombinant protein antigens. *Taq* polymerase-amplified rVP6 cDNA from gene 6 segment was cloned into TOPO TA^®^ pCRII vector as per manufacturer's instructions (Invitrogen, Carlsbad, CA). The inserted sequences were verified on an ABI automated DNA sequencer using dye-labeled terminator chemistry. The rVP6 DNAs were cloned into a shuttle vector (pDONR221) using the primers outlined in Table 1 and transferred into the baculovirus expression vector (pDEST17), using the Gateway^®^ PCR cloning system (Invitrogen). After each gene transfer, the sequences were verified. This expression cassette encoded an N-terminal hexa-histidine (His) tag to facilitate protein purification.

Recombinant baculovirus (rBV) encoding these VP6 genes were produced using the Bac-to-Bac^®^ baculovirus expression system (Invitrogen). The VP6 cDNA expression cassette was transferred from pDEST17 into the baculovirus shuttle DNA (bacmid) by transposition within chemically competent *E. coli* DH10Bac™ cells. Cells containing the bacmid with inserted VP6 cDNA were selected by blue-white screening of cells on triple antibiotic (50 μg/ml kanamycin, 7 μg/ml gentamycin and 10 μg/ml tetracycline) agar plates and bacmid DNA was isolated and transfected into *Spodoptera frugiperda* (*Sf*9) insect cells to make new rBV encoding the VP6 gene (rBV-rVP6) fused to an N-terminal His tag. P1 stocks were isolated and the viral titers were determined by plaque assay and amplified by subsequent infections of insect cells at an MOI of 0.1.

**Fig. 1:**
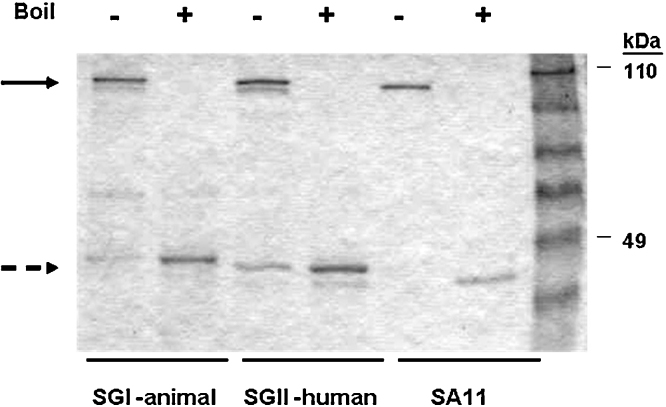
Analysis of recombinant VP6 proteins purified from *Sf*9 insect cells. Coomassie-blue stained gel analysis of purified rVP6 (*M*_wt_ ∼ 44 kDa) derived from an animal–human (SGI), human (SGII) or laboratory reference simian (SA11) VP6. RV VP6 fused to a N-terminal hexa-histidine tag was expressed in *Sf*9 cells infected with baculovirus encoding RV gene 6 and purified using Ni-NTA beads. Purified protein was treated ±100 °C for 5 min in denaturing buffer and analyzed by SDS-PAGE. The upper solid arrow indicates the VP6 trimers and the lower (broken) arrow indicates the monomeric form.

His-tagged rVP6 proteins were expressed in insect cells using the baculovirus expression system. Spinner flasks containing 3.5 × 10^6^ *Sf*9 insect cells per 200 ml of Grace's insect cell medium containing 0.5% fetal bovine sera (FBS, HyClone, Logan, UT) were infected with either rBV-rVP6 virus at a multiplicity of infection of 5. At 3 days post infection, the cell suspensions were centrifuged at 2000 × *g* for 10 min and cell pellets were washed with PBS. Cell contents were extracted using 10 mM sodium phosphate buffer containing 300 mM NaCl, 20 mM imidazole, 25 mM triethanolamine and 1% Sarcosyl (lysis buffer) containing protease inhibitors (aprotinin, leupeptin, and pepstatin (Sigma, St. Louis, MO.) at 1 μg/ml each). His-tagged rVP6 protein was then purified on nickel-nitrilotriacetic acid (Ni-NTA-agarose) (Qiagen, Valencia, CA.) according to the manufacturer's instructions. The protein concentration of rVP6 was determined by the Bradford protein assay (Bio-Rad, Hercules, CA) microtiter plate method. Purity was assessed by staining sodium dodecyl sulfate–polyacrylamide gels (SDS–PAGE) of denatured protein with Coomassie stain (Fig. 1).

The native VP6 protein forms a trimer on the viral capsid (Prasad et al., 1988) and the rVP6 products resemble the native conformation when run on a denaturing gel as has been previously described (Estes et al., 1987; Gorziglia et al., 1988; Petitpas et al., 1998). Following boiling, the monomeric form predominates. The molecular weight of the SGI and SGII monomers corresponds to the predicted molecular weight of VP6 (44 kDa). The expressed proteins were then analyzed by immunoblotting with a rabbit anti-rotavirus polyclonal sera. Western blotting showed that the polyclonal sera reacted specifically against the monomeric and trimeric rVP6 indicating that SGI and SGII were the antigenic protein.

DELFIA employs the lanthanide chelate europium (Eu^3+^)-labeled secondary antibodies which possess a high fluorescence intensity and virtually no background resulting in a highly sensitive detection method (Siitari et al., 1983). The resultant wider assay dynamics enabled us to analyze a large panel of serum samples at a single serum dilution (1/100) using less specimen volume than ELISA thus making it more suitable for high throughput surveys where small volumes of sample (e.g. neonatal serum) are available. The DELFIA reagents and Victor plate reader were purchased from Perkin Elmer (Waltham, MA). Unless otherwise stated, the assay volumes used were 75 μl and the assay diluent and wash buffer consisted of 10 mM Tris–HCl, pH 7.0, containing 0.05% Tween 20 (TBS-T). Black 96-well Fluotrac 200 medium binding microtiter plates (Greiner Bio One, Monroe, NC) were coated with either SGII-human or SGI-animal rVP6 (10 μg/ml) in 0.1 M sodium carbonate buffer pH 9.6 overnight at 4 °C. Plates were blocked with 400 μl of 5% w/v non-fat dried milk in TBS (Blotto) for 2 h at 37 °C in a humidified chamber. Following washing, human sera samples at a dilution of 1/100 in 10% blotto were added to antigen-coated and blank wells for 2 h at 37 °C. Following washing, plates were incubated with monoclonal mouse anti-human anti-IgG-labeled Eu^3+^ (1/500) in DELFIA buffer overnight at 4 °C followed by the addition of 100 μl of DELFIA enhancement buffer to each well. The plate was shaken gently for 30 min at room temperature to allow dissociation of the fluorescent lanthanide chelates. The fluorescence was read using the Eu^3+^ time-resolved fluorescence program set in the VICTOR^2^ multi-label plate reader.

The assay used to measure serum anti-RV IgG antibodies is a modification of an ELISA described previously (Ward et al., 1989). Briefly, 96-well microtiter plates (Corning Costar, Lowell, MA) were coated overnight with purified rabbit anti-rotavirus IgG at 4–8 °C. The plates were washed with phosphate buffered saline containing 0.05% Tween-20. Fifty microliters of WC3 virus lysate or mock-infected MA104 cell lysate were added to duplicate wells. A series of 2-fold dilutions of a standard serum pool was tested in each run to generate a standard curve. The standard serum pool was assigned arbitrary concentrations of 5000 U of IgG/ml. Serum samples were diluted 1 in 200 in 1% bovine serum albumin (BSA) as diluent (Invitrogen) or tested in higher dilutions if derived IgG values were outside the range of the standard curve. Anti-rotavirus IgG in the samples was detected using a biotin-conjugated anti-human IgG secondary antibody (Jackson ImmunoResearch Laboratories, Inc., West Grove, PA), peroxidase-conjugated avidin–biotin (Vector Laboratories Inc., Burlingame, CA) and o-phenylene diamine (OPD) substrate (Sigma). The plates were read at 492 nm. The OD value for each sample was generated by subtraction of the average optical density (OD) of the duplicate MA104 cell lysate wells from the average OD of sample in wells coated with virus lysate.

IgG response to rVP6 as measured by DELFIA was compared to the ELISA response. Although VP6 IgA is more widely used in clinical studies, IgG antibody responses were chosen to compare DELFIA and ELISA. This was based mainly on the fact that a large number of serum samples were collected at 6 monthly intervals wherein IgG is a more suitable marker for analysis. It has been identified as the most reliable and consistent marker for seroconversion in patients 1–30 months of age (Xu et al., 2005).

Serum samples from the birth cohort were divided into two groups–(i) RV infection detected in stool and serum samples showed a ≥2-fold increase in IgG levels within a 90 day period of an RV positive stool (“Inf < 90 days”) or (ii) RV infections identified only by serology; serum samples showed a ≥2-fold increase in IgG levels but no RV was detected in stool within a 90 day period (“No Inf < 90 days”). The choice of ≥two-fold as a measure of seroconversion was based on the fact that children in this cohort had a high frequency of RV infections resulting in maintenance of high levels of antibodies in circulation and therefore, the boost in immune response after an infection may not be very striking. The ability of DELFIA to detect ≥2-fold increase in IgG levels in the “Inf < 90 days” group (29%) was comparable to the ELISA (23%). The ELISA (19%) and DELFIA (16%) showed similar performance when analyzing the samples collected in the “No Inf < 90 days” group. When the criteria for serological detection of RV infection was increased to ≥3-fold or 4-fold increase in serum VP6 IgG levels, the DELFIA (3-fold = 21%; 4-fold = 19%) and ELISA (3-fold = 22%; 4-fold = 20%) performances were comparable in the “Inf < 90 days” group. Similar results were seen for samples collected in the “No Inf < 90 days” group between ELISA (3-fold = 14%; 4-fold = 11%) and DELFIA (3-fold = 10%; 4-fold = 8%). The concordance between DELFIA and ELISA, irrespective of what serum fold-rise was analyzed, was highly significant (*p* < 0.05) in both groups (Table 2).

In conclusion this study demonstrates that an immunoassay using recombinant VP6 can give equivalent results as the virus-capture ELISA. The rVP6 DELFIA is less-labor intensive, uses lower volume of clinical sample, requires only a single test dilution and is thus more suitable for highthrougput screening in clinical studies. Another advantage of DELFIA is its demonstrated ability to simultaneously measure virus-specific serum IgG and IgA (Kavanagh et al., 2011) which would improve the usefulness of the rVP6 assay further. Additionally, recombinant protein is safer and easier to produce in high quantities than virus lysate. Based on the success of the recombinant VP6 studies, the antibody responses generated to the recombinant viral capsid proteins VP7 and VP4 as well as the enterotoxin NSP4 are currently under evaluation in order to identify a possible correlate of protection to RV-induced disease.

## Conflict of interest statement

There are no reported conflicts of interest between authors or the author's institution with other people or organizations within 3 years of the beginning of the submitted work that may inappropriately influence the author's work.

## Role of the funding source

This work was funded by The Wellcome Trust Trilateral Initiative for Infectious Diseases (grant no. 063144). The funding source had no role, in study design; in the collection, analysis, and interpretation of data; in the writing of the report; and in the decision to submit the paper for publication.

## Figures and Tables

**Table 1:** Primer sequences used for amplifying RV VP6 genes.

VP6	SGII forward 5′-ATG GAG GTT CTG TAC TCA C-3′
VP6	SGI forward 5′-ATG GAT GTC CTG TAC TCC TTG TCA AAA ACT-3′
VP6	SGII and SGI reverse 5′-**CTA** GGT CAC ATC CTC TCA CTA C-3′

Start (underlined) and stop (bold) codons precede the VP6-specific sequences.

**Table 2:** Comparison of DELFIA and ELISA performance.

Stool RV detection	Fold change	DELFIA (%)	ELISA (%)	*p*-Value
Inf < 90 days	2-fold	29	23	<0.001
	3-fold	21	22	<0.001
	4-fold	19	20	<0.001

No Inf < 90 days	2-fold	16	19	<0.001
	3-fold	10	14	0.02
	4-fold	8	11	0.01

The ability of DELFIA or ELISA to detect RV infection as defined as a minimum ≥2-fold rise in serum VP6 IgG levels between consecutive serum samples was analyzed. A panel of serum from children enrolled in a birth cohort study was used to perform the analysis. Specimens were categorized into groups where an RV infection was detected within a 90 days period (“Inf < 90 days”) or if no RV infection was detected within the same timeframe (“No Inf < 90 days”). For the “Inf < 90 days” group 112 sera were screened and for the “No Inf < 90 days” 124 were analyzed. Comparative analysis of DELFIA and ELISA was carried out using the Chi-squared test where *p* < 0.05 indicates a strong association between the two assays.
